# Association of Dysphagia and Oral Involvement with Growth and Nutritional Status in Pediatric Lipoid Proteinosis: A Prospective Cross-Sectional Study

**DOI:** 10.3390/nu18203439

**Published:** 2026-10-20

**Authors:** Demet Teker-Duztas, İsa An

**Affiliations:** 1 Department of Pediatric Gastroenterology, Ankara Etlik City Hospital, 06170 Ankara, Türkiye; 2 Department of Pediatric Gastroenterology, Şanlıurfa Training and Research Hospital, 63040 Şanlıurfa, Türkiye; 3 Department of Dermatology, Şanlıurfa Training and Research Hospital, 63040 Şanlıurfa, Türkiye; isaan1989@gmail.com

**Keywords:** lipoid proteinosis, malnutrition, nutritional status, dysphagia

## Abstract

Background/Objectives: Lipoid proteinosis (LP) is a rare autosomal recessive genodermatosis characterized by oral and lingual involvement, frequently accompanied by dysphagia and recurrent oral ulcers. The relationships between these clinical manifestations and nutritional status or growth have not been clearly established. This study evaluated nutritional, growth, and micronutrient statuses in pediatric patients with LP and investigated their associations with dysphagia, oral ulcer frequency, and a composite oral involvement score. Methods: This prospective, cross-sectional, single-center study enrolled 30 pediatric patients with LP. Anthropometric measurements, laboratory results, and clinical data regarding oral involvement and dysphagia were collected. A study-specific composite oral involvement score (range: 0–4) was calculated based on restricted mouth opening, restricted tongue movement, presence of a thick or woody tongue, and active oral ulcers. Results: Mean age was 8.44 ± 4.97 years; 66.7% were male, and all had parental consanguinity. Mean HFA and WFH/BMI z-scores were −1.44 ± 1.07 and −0.55 ± 1.28; stunting and wasting occurred in 26.7% and 6.7%. Active oral ulcers and dysphagia were present in 70.0% and 60.0%. Iron-deficiency anemia (33.3%) and vitamin D deficiency (16.7%) were the most common deficiencies. Patients with dysphagia had lower HFA z-scores (*p* < 0.001; Cohen’s *d* = 1.91), which remained significant after Holm correction. The association between dysphagia and stunting did not remain significant after correction, and no other associations were found with dysphagia, oral ulcer frequency, or the composite oral involvement score. Conclusions: Dysphagia was associated with lower HFA z-scores in pediatric LP, whereas oral ulcer frequency and composite oral involvement were not. These exploratory findings suggest that swallowing function should be assessed during follow-up; larger multicenter prospective studies with standardized swallowing assessments are needed.

## 1. Introduction

Lipoid proteinosis (LP) is a rare autosomal recessive genodermatosis caused by biallelic pathogenic or likely pathogenic variants in the extracellular matrix protein 1 (ECM1) gene, located on chromosome 1q21 [1]. LP was first described by Siebermann in 1908 [2], and its classic clinical presentation was delineated in 1929 by dermatologist Erich Urbach and otorhinolaryngologist Camillo Wiethe [3]. Histopathologically, LP is characterized by the deposition of periodic acid–Schiff (PAS)-positive, diastase-resistant amorphous hyaline material in the dermis, perivascular regions, and dermoepidermal junction, as well as thickening of the basement membrane [4]. Worldwide, fewer than 500 cases have been reported [5,6,7]. Most affected individuals are of Asian origin, with Türkiye accounting for the most reported cases [8]. No definitive treatment is currently available; management therefore focuses on symptom control and prevention of complications [9].

The earliest clinical manifestation of LP is typically hoarseness or a hoarse cry, which develops at birth or in early infancy due to hyaline material deposition in the laryngeal mucosa and vocal cords [10]. This is generally followed within the first two years of life by the appearance of waxy papules, bullous lesions, and acneiform scarring on the face, trunk, and extremities, as well as beaded papules along the eyelid margins (moniliform blepharosis), a characteristic feature of the disease [2,3]. Skin thickening and hyperkeratosis may occur at friction sites, including the elbows, knees, hands, and axillae [11]. Oral mucosal involvement often manifests as tongue induration, thickening of the sublingual frenulum with consequent restriction of tongue mobility, and cobblestone-like thickening of the lips, tongue, buccal mucosa, and gingiva [12]. Oral involvement may also present with recurrent ulcerations and transient swelling of the lips and tongue [13]. Infiltration of the oral mucosa and tongue by hyaline material may lead to xerostomia and dysphagia [14].

The literature on LP primarily comprises case reports and case series, with most studies focusing on dermatological manifestations and oral mucosal lesions [12,15,16,17,18,19]. Restricted mobility from hyaline material deposition in the oral mucosa and tongue, along with xerostomia, dysphagia, and recurrent oral ulcerations, may negatively affect dietary intake. Dysphagia is associated with inadequate energy and micronutrient intake by reducing oral intake, prompting avoidance of certain food consistencies, and preventing meal completion [20,21,22]. Similarly, oral ulcers, regardless of etiology, may limit dietary intake due to pain and feeding difficulties and may be bidirectionally related to micronutrient deficiencies [23]. However, to our knowledge, no study has systematically investigated the associations between oral oral involvement or dysphagia and nutritional status, growth parameters, or micronutrient levels in children with LP using standardized measures. This represents a significant gap in the current understanding of nutritional and growth outcomes in pediatric LP.

Given these considerations, the primary aim of this study was to prospectively evaluate nutritional status, growth parameters, and micronutrient levels in children and adolescents with LP. The secondary aim was to investigate associations between oral ulcer frequency or dysphagia and anthropometric measurements, growth indicators, and biochemical markers of nutritional status.

## 2. Materials and Methods

### 2.1. Study Design

This was a prospective, cross-sectional, single-center study. Approval was obtained from the Harran University Clinical Research Ethics Committee (approval date: 22 July 2024; approval number: HRÜ/24.10.28). All procedures adhered to the principles of the Declaration of Helsinki. Written informed consent was obtained from all participants or their legal guardians. All collected data were stored in accordance with confidentiality standards and used exclusively for scientific purposes.

### 2.2. Study Population

The study included patients under 18 years old who were seen at the Dermatology Outpatient Clinic of Şanlıurfa Training and Research Hospital and had a confirmed diagnosis of LP by clinical, histopathological, or genetic methods. Because LP is rare, we did not calculate a sample size in advance. Instead, we invited all eligible patients seen at the center during the study period to take part. Families of patients with LP were contacted by phone and informed about the study. Those who agreed were invited to the Pediatric Gastroenterology Outpatient Clinic for evaluation. All evaluations took place between 1 and 15 September 2024, and the same pediatric gastroenterologist and dermatologist assessed each patient.

Clinical confirmation was based on hoarseness or a weak, husky cry starting in infancy; bead-like papules along the eyelid margins (moniliform blepharosis); waxy papules on the face and limbs; acne-like scarring; wart-like plaques in areas exposed to friction; and thickened skin. Histopathological confirmation was based on finding periodic acid–Schiff (PAS)-positive, diastase-resistant hyaline material deposits in the dermis and around blood vessels [24]. All 30 patients fulfilled the clinical diagnostic criteria and had histopathological confirmation. Genetic testing was performed in 11 patients (36.7%), and biallelic pathogenic or likely pathogenic variants in the ECM1 gene (1q21) were identified in all of them [25]. In the other 19 patients, testing was not performed because of cost and limited access, and the diagnosis was based on clinical, histopathological, and family history findings.

Patients were excluded if they declined participation, had incomplete data, had a chronic condition that could affect nutritional status, or had another syndromic disorder. Siblings under 18 years of age were eligible. The 30 patients belonged to 23 families (1 family contributed 4 siblings, 4 families 2 each, and 18 families 1 patient; 12 patients [40.0%] were siblings from five families). Affected siblings aged ≥ 18 years were not enrolled.

**Figure 1:**
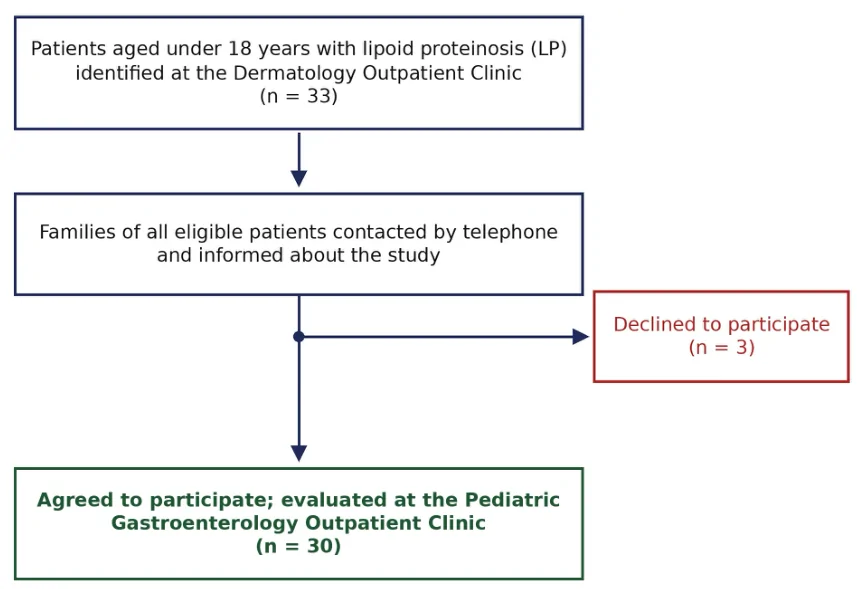
Study flowchart.

Figure 1 shows the patient selection and enrollment process. Three of the 33 eligible patients declined to participate, and no patient was excluded because of incomplete data, coexisting chronic conditions, or another syndromic disorder.

### 2.3. Data Collection

Demographic, clinical, and nutrition-related data were prospectively collected during the examination using a standardized data collection form. No therapeutic or nutritional interventions were administered during the study, and all analyses used enrollment data. To reduce measurement and information bias, the same investigators performed all anthropometric and clinical assessments, and a second investigator independently verified data entries. Parental consanguinity was recorded by parental interview and classified as first-degree (first cousins), second-degree (first cousins once removed), or third-degree (second cousins). Dietary intake, feeding duration, food consistency modifications, and dietary restrictions were not quantitatively assessed because families could not complete the food records requested; feeding difficulties were considered only qualitatively in defining dysphagia.

#### 2.3.1. Clinical Assessment

Oral mucosal findings were systematically documented by the examining pediatric gastroenterologist. In addition to recording the presence of active oral ulcers at examination, the ulcer frequency was recorded based on parent or caregiver reports. Patients who reported developing ulcers at intervals of ≤2 weeks were classified as having frequent ulcers, whereas those who reported ulcers at intervals of >2 weeks were classified as having rare ulcers. There is no established definition of ulcer frequency in LP; the ≤2-week cut-off reflects the recurrence interval most commonly reported by families and has not been validated.

**Figure 2:**
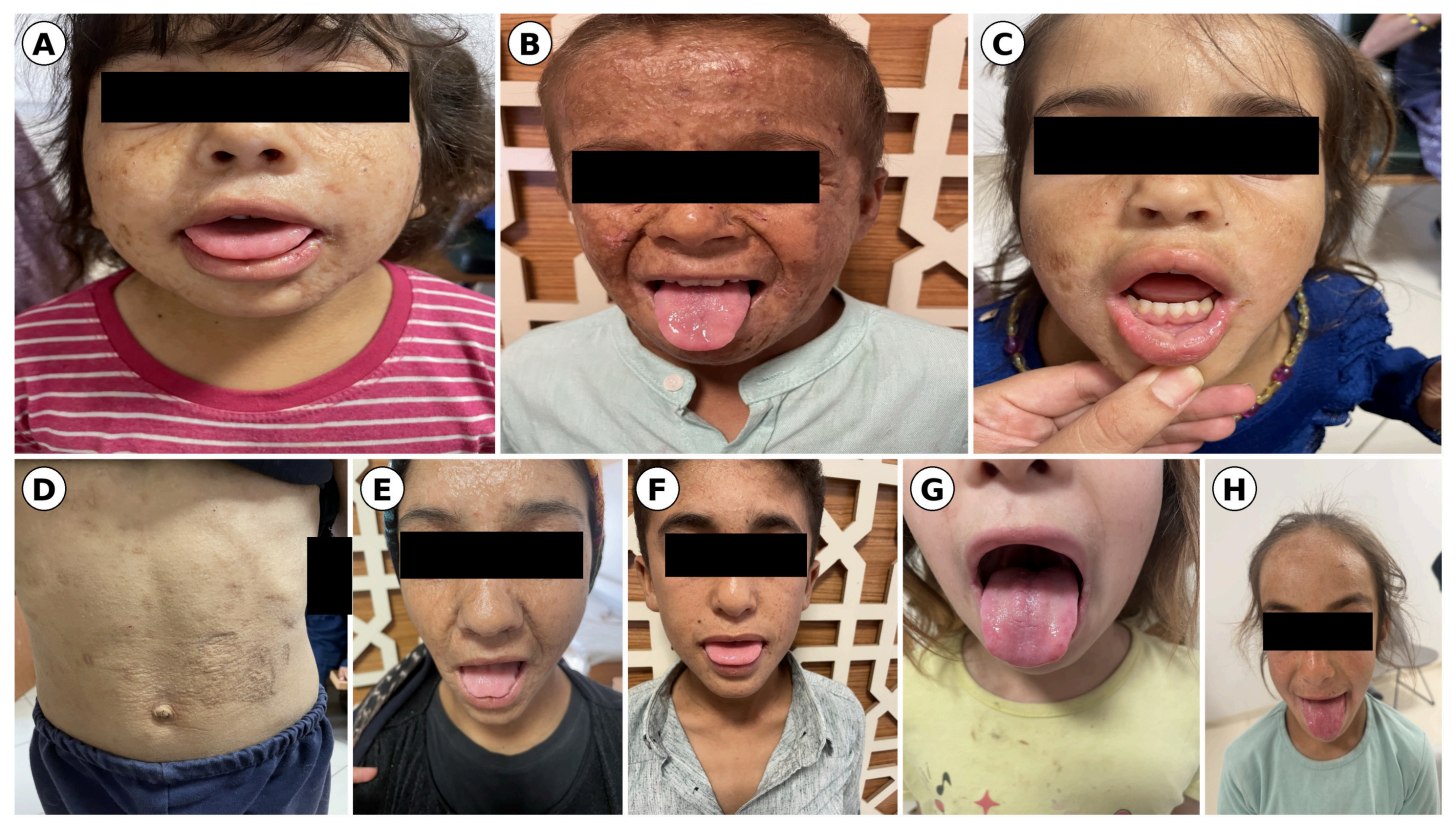
Representative clinical findings in children with lipoid proteinosis from the study cohort. (**A**–**C**,**E**–**H**) Facial and oral findings in seven patients: diffuse waxy, thickened and hyperpigmented facial skin with atrophic pitted scars (**A**–**C**,**E**,**F**); thickened, infiltrated lips (**A**,**C**); and a thickened tongue with limited protrusion (**A**–**C**,**E**–**H**). (**D**) Trunk of a further patient showing scattered hyperpigmented, hyperkeratotic plaques with scarring. Eyes are masked to protect patient identity. Written informed consent for publication of these images was obtained from the patients’ parents/legal guardians.

Oral involvement was assessed using a composite oral involvement score ranging from 0 to 4, with one point assigned for each of the following: restricted mouth opening, restricted tongue movement, thickened or woody appearance of the tongue, and active oral ulcers. In the absence of standardized numerical thresholds, mouth opening and tongue movement were classified by the examining pediatric gastroenterologist as either “restricted” or “normal,” and the tongue as either “thickened/woody” or “normal,” based on clinical judgment (Figure 2). Voice change (hoarseness) was recorded separately but was excluded from this score, as it was considered a functional indicator rather than a direct oral finding. The score was developed because oral involvement in LP comprises several characteristic findings and no validated scale exists. A simple additive score of binary clinical findings was chosen for feasibility. It is exploratory and study-specific, with equal weighting used because no established weighting exists; because it combines chronic structural findings with potentially transient ulcers, ulcer frequency was also analyzed separately.

Dysphagia was assessed by the same pediatric gastroenterologist through caregiver reports, clinical history, and physical examination, before anthropometric measurements were taken and z-scores calculated. The features considered were difficulty swallowing solid foods, prolonged feeding duration, coughing or choking during feeding, and refusal of oral intake. Each of the four features was asked about separately and recorded for every patient, and dysphagia was classified as “present” when at least three of the four features were evident and “absent” otherwise. Difficulty with solids and liquids was evaluated separately, and no instrumental swallowing assessment was performed.

#### 2.3.2. Anthropometric Assessment

Body weight was measured to the nearest 0.1 kg using a calibrated digital scale. For children younger than 2 years, recumbent length was measured with an infantometer. For children aged 2 years and older, standing height was measured using a stadiometer [26].

Nutritional status was evaluated using World Health Organization (WHO) growth standards. Height-for-age (HFA), weight-for-height (WFH), and body mass index (BMI)-for-age z-scores were calculated with WHO Anthro (version 3.2.2) and AnthroPlus (version 1.0.4) software. Consistent with WHO recommendations, WFH z-scores were applied to children younger than 5 years, while BMI-for-age z-scores were used for those aged 5 years and older [27].

Growth status was evaluated according to WHO recommendations [27]. Stunting was defined as an HFA z-score below −2 standard deviations (SDs) [27]. Wasting was defined as a z-score below −2 SDs for WFH in children younger than 5 years and for BMI-for-age in those aged 5 years and older [27]. WFH (<5 years) and BMI-for-age (≥5 years) are the WHO-recommended indicators of current weight status for their age groups, are standardized z-scores on the same scale, and share the same wasting cut-off (<−2 SDs); they were therefore combined into a single WFH/BMI z-score, which also avoided very small age-specific subgroups (9 patients < 5 years, 21 ≥ 5 years).

#### 2.3.3. Laboratory Assessment

Venous blood samples were obtained from all participants at the time of study enrollment. The hospital central biochemistry laboratory conducted analyses using automated analyzer systems, following the manufacturers’ protocols. Standard internal quality-control procedures were implemented for all tests.

Serum albumin concentration was measured to assess nutritional status. Hemoglobin (Hb), mean corpuscular volume (MCV), serum iron, total iron-binding capacity (TIBC), and ferritin were analyzed to evaluate iron metabolism and anemia. Anemia was defined according to World Health Organization (WHO) age-specific criteria [28]. A serum ferritin level < 15 μg/L was considered indicative of depleted iron stores [29]. Anemia and depleted iron stores were identified as iron deficiency anemia.

Serum folate, vitamin B12, and 25-hydroxyvitamin D concentrations were measured to evaluate micronutrient status. Folate deficiency was defined as a serum folate concentration of <4 ng/mL, and vitamin B12 deficiency as a serum vitamin B12 concentration of <203 pg/mL [30]. A serum 25(OH)D concentration of <20 ng/mL was classified as vitamin D deficiency [31].

Serum calcium, phosphorus, magnesium, alkaline phosphatase (ALP), and parathyroid hormone (PTH) concentrations were measured to assess bone and mineral metabolism. Serum glucose, urea, creatinine, alanine aminotransferase (ALT), aspartate aminotransferase (AST), and C-reactive protein (CRP) concentrations were also determined. All results were interpreted using the hospital laboratory’s age-specific reference ranges.

### 2.4. Statistical Analysis

Statistical analyses were performed using IBM SPSS Statistics for Windows, version 25.0 (IBM Corp., Armonk, NY, USA). Due to the small sample size, the Shapiro–Wilk test was used to assess the normality of continuous variables instead of the Kolmogorov–Smirnov test. Normality was evaluated separately within each subgroup defined by dysphagia status and oral ulcer frequency. For descriptive analyses, continuous variables were reported as mean ± standard deviation (SD) when normally distributed, and as median and interquartile range (IQR) when non-normally distributed. Categorical variables were summarized as frequencies and percentages (*n*, %).

For group comparisons based on dysphagia status (present or absent) and oral ulcer frequency (frequent or rare), normally distributed continuous variables were reported as means with 95% confidence intervals (CIs) and compared using the independent-samples *t*-test. If Levene’s test indicated unequal variances (*p* < 0.05), Welch’s *t*-test was applied. Non-normally distributed continuous variables were reported as medians and IQRs and compared using the Mann–Whitney *U* test. Associations between categorical variables were assessed using Pearson’s chi-square (χ^2^) test or Fisher’s exact test when the expected cell count in a 2 × 2 table was less than 5. Because multiple group comparisons were performed (27 for dysphagia status and 27 for oral ulcer frequency), Holm correction was additionally applied within each set of comparisons; unadjusted *p* values are presented in the tables, and only findings that remained significant after Holm correction were interpreted as statistically significant.

Effect sizes were calculated for all group comparisons. Cohen’s *d* was used for independent-samples *t*-tests, the effect size *r* (*r* = *Z*/√*N*, where *Z* is the standardized test statistic) for Mann–Whitney *U* tests, and Cramér’s *V* for chi-square and Fisher’s exact tests. In 2 × 2 tables, Cramér’s *V* is equivalent to the phi coefficient. For *d* and *r*, a positive sign indicates higher values in the first-listed group in each table. Effect sizes were interpreted according to Cohen’s recommended thresholds: values of approximately 0.10, 0.30, and 0.50 indicated small, medium, and large effects, respectively, for *r* and *V*, while values of approximately 0.20, 0.50, and 0.80 indicated small, medium, and large effects, respectively, for *d*.

To evaluate the relationship between composite oral involvement and indicators of nutritional status and growth, associations of the composite oral involvement score (a discrete ordinal scale ranging from 0 to 4) with age, anthropometric z-scores (HFA and WFH/BMI), and nutrition-related laboratory parameters (albumin, iron, TIBC, ferritin, 25-hydroxyvitamin D, vitamin B12, folate, and hemoglobin) were assessed using Spearman’s rank correlation coefficient (rho), due to the ordinal or discrete nature of the variables and the small sample size. No multiple-comparisons correction was applied; therefore, these findings were interpreted as hypothesis-generating rather than confirmatory.

Given the small sample size and the large number of comparisons, all analyses were considered exploratory. Multivariable regression was not performed because HFA z-scores are already standardized for age and sex, the sample size (*n* = 30) allowed only very few covariates to be included without overfitting. A two-sided *p* value of less than 0.05 was considered statistically significant in all analyses.

## 3. Results

The study included 30 pediatric patients with LP (Table 1). The majority were male (66.7%), with a mean age of 8.44 years (SD = 4.97; range = 0.98–17.94). All families reported parental consanguinity (100.0%), most commonly between first cousins (93.3%).

Anthropometric assessment showed a mean HFA z-score of −1.44 (SD = 1.07) and a mean WFH/BMI-for-age z-score of −0.55 (SD = 1.28), indicating a tendency toward growth impairment across the cohort. Stunting was present in 26.7% of patients, whereas wasting was present in only 6.7%.

Regarding oral and mucosal involvement, active oral ulcers were present in 70.0% of patients, and ulcer recurrence was frequent (≤2 weeks) in 63.3% of cases. Voice change, restricted mouth opening, restricted tongue movement, and thick/woody tongue were observed in 43.3%, 46.7%, 53.3%, and 56.7% of patients, respectively. Dysphagia was present in more than half of the cohort (60.0%); all affected patients had difficulty with solid foods only, and none had difficulty with liquids.

Laboratory and hematological findings are summarized in Table 2.

Iron-deficiency anemia was identified in 10 patients (33.3%), vitamin D deficiency in 5 (16.7%), vitamin B12 deficiency in 1 (3.3%), and folate deficiency in 1 (3.3%).

Group comparisons according to dysphagia status are presented in Table 3. At the unadjusted 0.05 level, patients with dysphagia had lower HFA z-scores (M = −2.04, 95% CI [−2.45, −1.64]) than patients without dysphagia (M = −0.55, 95% CI [−1.01, −0.09]), t(28) = 5.12, *p* < 0.001, *d* = 1.91. Hemoglobin concentrations were also lower in the dysphagia group (M = 11.25, 95% CI [10.48, 12.02]) than in the group without dysphagia (M = 12.66, 95% CI [11.56, 13.76]), t(28) = 2.33, *p* = 0.027, *d* = 0.87. The calcium distribution differed between groups (Mdn = 9.48 vs. 9.58 mg/dL; exact *p* = 0.048), and stunting was more frequent among patients with dysphagia (44.4% vs. 0.0%; Fisher’s exact *p* = 0.010). After Holm correction across the 27 dysphagia-related comparisons, only the difference in HFA z-score remained statistically significant; the other findings should therefore be interpreted as exploratory.

**Table 3:** Comparison of Sociodemographic, Clinical, Anthropometric, and Laboratory Characteristics by Dysphagia Status.

Variable	No Dysphagia (*n* = 12)	Dysphagia (*n* = 18)	Test Statistic	*p*	Effect Size
**Clinical characteristics**					
Sex, male ^a^	8 (66.7)	12 (66.7)	0.00 ^χ2^	1.000	*V* = 0.00
Age, years	8.17 [4.35, 11.98]	8.62 [6.47, 10.77]	−0.24 ^t^	0.812	*d* = −0.09
HFA z-score	−0.55 [−1.01, −0.09]	−2.04 [−2.45, −1.64]	5.12 ^t^	**<0.001**	*d* = 1.91
WFH/BMI z-score ^b^	−0.40 (1.54)	−0.95 (2.10)	87.0 ^U^	0.391	*r* = 0.16
Stunting ^a^	0 (0.0)	8 (44.4)	7.27 ^χ2^	0.010	*V* = 0.49
Wasting ^a^	1 (8.3)	1 (5.6)	0.09 ^χ2^	1.000	*V* = 0.05
Oral ulcer frequency, frequent (≤2 weeks) ^a^	9 (75.0)	10 (55.6)	1.17 ^χ2^	0.442	*V* = 0.20
Voice change	7 (58.3)	6 (33.3)	1.83 ^χ2^	0.176	*V* = 0.25
Restricted mouth opening	4 (33.3)	10 (55.6)	1.43 ^χ2^	0.232	*V* = 0.22
Restricted tongue movement	5 (41.7)	11 (61.1)	1.09 ^χ2^	0.296	*V* = 0.19
Thick/woody tongue	5 (41.7)	12 (66.7)	1.83 ^χ2^	0.176	*V* = 0.25
**Laboratory parameters**					
Glucose, mg/dL	92.92 [83.04, 102.79]	90.41 [84.92, 95.89]	0.52 ^t^	0.608	*d* = 0.19
Calcium, mg/dL ^b^	9.58 (0.45)	9.48 (0.42)	61.5 ^U^	0.048	*r* = 0.36
Magnesium, mg/dL	2.10 [2.04, 2.16]	2.05 [1.99, 2.12]	1.18 ^t^	0.249	*d* = 0.44
Phosphorus, mg/dL	4.40 [3.94, 4.86]	4.52 [4.27, 4.78]	−0.53 ^t^	0.598	*d* = −0.20
ALP, U/L ^c^	213.74 [160.38, 267.10]	183.00 [163.20, 202.80]	1.18 ^t^	0.256	*d* = 0.50
PTH, pg/mL ^b^	32.05 (9.27)	30.01 (13.32)	88.0 ^U^	0.415	*r* = 0.15
Vitamin D, ng/mL ^c^	31.56 [27.21, 35.92]	30.50 [24.03, 36.97]	0.29 ^t^	0.772	*d* = 0.10
Albumin, g/L	45.70 [44.43, 46.97]	44.36 [43.25, 45.47]	1.68 ^t^	0.105	*d* = 0.62
CRP, mg/L ^b^	0.43 (2.89)	0.81 (2.87)	130.0 ^U^	0.368	*r* = −0.17
Hemoglobin, g/dL	12.66 [11.56, 13.76]	11.25 [10.48, 12.02]	2.33 ^t^	0.027	*d* = 0.87
MCV, fL ^b^	78.20 (8.18)	74.70 (17.98)	75.0 ^U^	0.172	*r* = 0.26
Iron, µg/dL	53.74 [32.86, 74.62]	44.73 [33.16, 56.31]	0.88 ^t^	0.386	*d* = 0.33
TIBC, µg/dL	329.56 [289.78, 369.34]	333.48 [290.49, 376.48]	−0.14 ^t^	0.894	*d* = −0.05
Ferritin, ng/mL	32.71 [19.88, 45.53]	35.24 [24.30, 46.18]	−0.32 ^t^	0.752	*d* = −0.12
Vitamin B12, pg/mL	405.80 [324.88, 486.72]	519.09 [420.48, 617.71]	−1.75 ^t^	0.091	*d* = −0.65
Folate, ng/mL ^b^	6.04 (2.63)	6.33 (3.49)	109.0 ^U^	1.000	*r* = −0.01

Notes: Values are Mean [95% confidence interval] for normally distributed continuous variables (independent-samples *t* test) and Median (interquartile range) for non-normally distributed continuous variables (Mann–Whitney test), based on Shapiro–Wilk results. HFA = height-for-age; WFH = weight-for-height; BMI = body mass index; ALP = alkaline phosphatase; CRP = C-reactive protein; TIBC = total iron-binding capacity; PTH = parathyroid hormone; MCV = mean corpuscular volume. *t* = independent-samples *t* test; *U* = Mann–Whitney *U* test; χ^2^ = Pearson chi-square test. Effect sizes: Cohen’s *d* for *t* tests, *r* for Mann–Whitney tests, and Cramér’s V (equivalent to φ in 2 × 2 tables) for categorical comparisons. Posi-tive d and r values indicate higher values in the first-listed group (No Dysphagia). *p* values are unadjusted; bold indicates significance after Holm correction across 27 comparisons. ^a^ *p* values obtained from Fisher’s exact test because expected cell counts were below 5; the χ^2^ values shown are Pearson χ^2^ statistics. ^b^ Mann–Whitney *U* test with exact two-sided significance; *U* is the statistic for the second-listed group, and *r* = *Z*/√*N* (*Z* = standardized test statistic). ^c^ Welch’s *t* test (equal variances not assumed) reported because Levene’s test was significant. Table 4 presents comparisons by oral ulcer frequency (frequent, ≤2 weeks, *n* = 19; rare, >2 weeks, *n* = 11). No continuous or categorical variable differed significantly between the groups at the unadjusted 0.05 level; consequently, no result was significant after Holm correction across the 27 comparisons. A medium-sized difference in WFH/BMI z-score (Mdn = −0.08 in the frequent group vs. −1.01 in the rare group; exact *p* = 0.057, *r* = 0.35) did not reach statistical significance.

**Table 4:** Comparison of Sociodemographic, Clinical, Anthropometric, and Laboratory Characteristics by Oral Ulcer Frequency.

Variable	Frequent (*n* = 19)	Rare (*n* = 11)	Test Statistic	*p*	Effect Size
**Demographic, anthropometric, and clinical characteristics**					
Sex, male ^a^	13 (68.4)	7 (63.6)	0.07 ^χ2^	1.000	*V* = 0.05
Age, years	8.53 [6.01, 11.04]	8.28 [5.08, 11.47]	0.13 ^t^	0.895	*d* = 0.05
HFA z-score	−1.31 [−1.88, −0.73]	−1.68 [−2.21, −1.15]	0.93 ^t^	0.362	*d* = 0.35
WFH/BMI z-score ^b^	−0.08 (1.89)	−1.01 (0.84)	60.0 ^U^	0.057	*r* = 0.35
Stunting ^a^	4 (21.1)	4 (36.4)	0.84 ^χ2^	0.417	*V* = 0.17
Wasting ^a^	1 (5.3)	1 (9.1)	0.16 ^χ2^	1.000	*V* = 0.07
Dysphagia ^a^	10 (52.6)	8 (72.7)	1.17 ^χ2^	0.442	*V* = 0.20
Voice change ^a^	7 (36.8)	6 (54.5)	0.89 ^χ2^	0.454	*V* = 0.17
Restricted mouth opening	11 (57.9)	3 (27.3)	2.63 ^χ2^	0.105	*V* = 0.30
Restricted tongue movement	9 (47.4)	7 (63.6)	0.74 ^χ2^	0.389	*V* = 0.16
Thick/woody tongue ^a^	10 (52.6)	7 (63.6)	0.34 ^χ2^	0.708	*V* = 0.11
**Laboratory parameters**					
Glucose, mg/dL ^b^	91.80 (19.40)	86.00 (18.00)	85.0 ^U^	0.420	*r* = 0.15
Calcium, mg/dL ^b^	9.57 (0.43)	9.52 (0.46)	81.0 ^U^	0.328	*r* = 0.18
Magnesium, mg/dL	2.05 [1.99, 2.11]	2.11 [2.04, 2.18]	−1.31 ^t^	0.202	*d* = −0.49
Phosphorus, mg/dL ^b^	4.73 (0.55)	4.48 (0.73)	77.0 ^U^	0.250	*r* = 0.22
ALP, U/L	197.15 [161.95, 232.35]	192.09 [165.93, 218.25]	0.21 ^t^	0.834	*d* = 0.08
PTH, pg/mL ^b^	32.00 (12.72)	30.46 (8.20)	106.0 ^U^	0.966	*r* = −0.01
Vitamin D, ng/mL	30.79 [25.03, 36.55]	31.16 [25.03, 37.29]	−0.09 ^t^	0.930	*d* = −0.03
Albumin, g/L	45.11 [44.04, 46.17]	44.54 [43.01, 46.07]	0.67 ^t^	0.506	*d* = 0.26
CRP, mg/L ^b^	0.77 (2.85)	0.51 (3.23)	93.0 ^U^	0.641	*r* = 0.09
Hemoglobin, g/dL	12.01 [11.10, 12.92]	11.47 [10.49, 12.45]	0.81 ^t^	0.423	*d* = 0.31
MCV, fL ^b^	75.40 (11.60)	75.60 (9.20)	110.0 ^U^	0.832	*r* = −0.04
Iron, µg/dL	49.21 [35.41, 63.01]	46.83 [29.21, 64.45]	0.23 ^t^	0.823	*d* = 0.09
TIBC, µg/dL	332.61 [296.95, 368.27]	330.72 [273.81, 387.63]	0.06 ^t^	0.950	*d* = 0.02
Ferritin, ng/mL	33.59 [23.62, 43.57]	35.32 [20.27, 50.37]	−0.21 ^t^	0.832	*d* = −0.08
Vitamin B12, pg/mL ^b^	403.60 (251.70)	424.40 (184.40)	109.0 ^U^	0.866	*r* = −0.04
Folate, ng/mL ^b^	6.21 (2.74)	6.32 (5.09)	125.0 ^U^	0.395	*r* = −0.16

Notes: Values are Mean [95% confidence interval] for normally distributed continuous variables (independent-samples *t* test) and Median (interquartile range) for non-normally distributed continuous variables (Mann–Whitney test), based on Shapiro–Wilk results. Frequent = oral ulcer recurrence ≤2 weeks; Rare = oral ulcer recurrence >2 weeks. HFA = height-for-age; WFH = weight-for-height; BMI = body mass index; ALP = alkaline phosphatase; CRP = C-reactive protein; TIBC = total iron-binding capacity; PTH = parathyroid hormone; MCV = mean corpuscular volume. *t* = independent-samples *t* test; *U* = Mann–Whitney *U* test; χ^2^ = Pearson chi-square test. Effect sizes: Cohen’s *d* is reported for *t* tests, *r* for Mann–Whitney tests, and Cramér’s *V* (equivalent to φ in 2 × 2 tables) for categorical comparisons. Positive d and r values indicate higher values in the first-listed group(No Dysphagia). *p* values are unadjusted; bold indicates significance after Holm correction across 27 comparisons. ^a^ *p* values obtained from Fisher’s exact test because expected cell counts were below 5; the χ^2^ values shown are Pearson χ^2^ statistics. ^b^ Mann–Whitney *U* test with exact two-sided significance; *U* is the statistic for the second-listed group, and *r* = *Z*/√*N* (*Z* = standardized test statistic).

The composite oral involvement score was 0 in 2 patients (6.7%), 1 in 9 (30.0%), 2 in 6 (20.0%), 3 in 5 (16.7%), and 4 in 8 (26.7%).

The relationship between the composite oral involvement score and age, anthropometric z-scores, and nutrition-related laboratory parameters was examined using Spearman’s rank-order correlations (Table 5). No correlation reached statistical significance (all *p* > 0.05).

## 4. Discussion

To the best of our knowledge, this is the first study to evaluate the associations of dysphagia and oral ulcer frequency with nutritional status, growth measures, and laboratory findings in pediatric patients diagnosed with LP; additionally, a composite oral involvement score was developed, and the associations of this score with growth and laboratory parameters were examined. The main finding was that dysphagia was strongly and clinically meaningfully associated with a lower HFA z-score, which remained significant after Holm correction, and with a higher frequency of stunting, which did not remain significant after adjustment for multiple comparisons. In contrast, no statistically significant association was found between oral ulcer frequency or the composite oral involvement score and growth or laboratory parameters. These results suggest that impaired swallowing function may be associated with growth in children with LP.

LP is an extremely rare disease. Laryngeal involvement and associated hoarseness are among the characteristic findings in the early stages of the disease [15,32]. Although studies have described the characteristics and severity of oral and laryngeal involvement [8,12,33,34], data on the relationship between these findings and nutritional status and growth remain limited. To the best of our knowledge, our study is one of the few to quantitatively evaluate the relationship between dysphagia and growth and nutritional indicators in pediatric patients with LP. Similar associations between swallowing difficulties and impaired growth have been described in other rare diseases with oral and esophageal involvement. In epidermolysis bullosa, oral blistering and scarring (microstomia, ankyloglossia) and esophageal stenosis restrict food intake, dysphagia typically begins with solid foods, and growth retardation is most frequent in the severe recessive dystrophic and junctional forms [35], in a retrospective Korean cohort of 30 children with EB, 56% were below the 3rd percentile for weight at last follow-up [36]. These findings parallel the solid-food dysphagia and impaired linear growth observed in our cohort, although the underlying mechanisms differ and these studies were largely descriptive, which limits direct comparison.

The lower HFA z-score and higher frequency of stunting among patients with dysphagia suggest that dysphagia may be associated with linear growth. The lack of a significant difference in WFH/BMI z-score between groups suggests the association may be more apparent in indicators of linear growth than in current body weight. However, because the study is cross-sectional, these findings do not establish the direction or causality of the relationship between dysphagia and growth. Although cases of short stature accompanying LP have been reported in the literature [37,38,39], the common underlying mechanism has not yet been clarified. An important aspect of our study is that effect sizes were evaluated in addition to statistical significance. However, because 12 of the 30 patients were siblings from five families, the observations were not fully independent. Siblings share genetic background and household environment, so familial clustering may have reduced the effective sample size, led to underestimated standard errors and overly optimistic *p* values, and partly accounted for the association between dysphagia and lower HFA z-scores. The statistical significance of this association (*p* < 0.001) may therefore be overstated and should be interpreted with caution. The large effect sizes observed in the associations of dysphagia with HFA z-score support the potential clinical importance of these findings. Nevertheless, larger longitudinal studies should confirm these results.

Another notable finding of the study was the absence of a statistically significant association between oral ulcer frequency or the composite oral involvement score and growth or laboratory parameters. Treatment of LP is symptomatic [40], and although various treatments have been proposed in the literature, the risk of recurrence of clinical findings has been reported to be high some time after completion of treatment [41,42,43]. The episodic course of oral lesions, their regression with treatment, or patients’ adaptation over time to oral involvement and restricted tongue movement may partially explain this result. However, these possibilities were not directly evaluated in our study. Given the limited sample size and multiple comparisons, these analyses are exploratory and may have lacked the power to detect weaker associations; the equal weighting of chronic structural findings and potentially transient ulcers may also have obscured associations of individual components. Therefore, these results do not indicate that oral lesions are unrelated to nutrition, but rather that the study could not demonstrate a significant association between the oral involvement measures used and growth or laboratory parameters. Our findings indicate that swallowing function, along with oral findings, should be considered when evaluating nutrition and growth in children with LP. Standardized swallowing outcome measures are important in patients with oropharyngeal dysfunction; a systematic review in adults treated for oropharyngeal cancer reported heterogeneous swallowing assessment tools that prevented pooling of results [44]. In clinical practice, children with LP and suspected dysphagia may benefit from a multidisciplinary evaluation involving pediatric gastroenterologists, otolaryngologists, swallowing specialists, and dietitians, allowing earlier recognition of swallowing problems and growth faltering. This suggests that standardized pediatric swallowing outcome measures with a multidisciplinary team would also be valuable in future studies of LP.

One of the main limitations of our study was that dysphagia was classified clinically from caregiver reports, history, and examination, using a simple count of clinical features (at least three of four) that has not been validated, and without instrumental assessment (fiberoptic endoscopic evaluation of swallowing or videofluoroscopic swallowing study); misclassification therefore cannot be excluded, and subjectivity and recall bias may have influenced the results. The same clinician later measured growth, without formal blinding. As a result, oral, pharyngeal, laryngeal, and esophageal involvement could not be differentiated, although hyaline deposition in these regions has been reported [45,46], and only solid-food dysphagia was observed, so our findings cannot be extended to liquids.

Other limitations relate to the oral assessments: a single examiner evaluated dysphagia status, ulcer frequency, structural oral findings, and the composite oral involvement score, so inter-rater reliability could not be assessed. The composite score has not been validated and gives equal weight to chronic structural findings and potentially transient ulcers and the ulcer frequency cut-off (≤2 weeks), based on the interval most commonly reported by families and on caregiver reports, is also non-validated and may be affected by recall bias. The single-center, cross-sectional design precludes causal inference and the comparison of periods with and without active ulcers, there was no control group, and dietary intake could not be assessed because families were unable to keep food records. Finally, the small sample, the large number of comparisons (correlations with the composite score were not adjusted for multiple testing), the absence of multivariable adjustment, and the inclusion of 12 siblings from five families, whose non-independent observations may have led to underestimated *p*-values and could not be adjusted for, mean that our analyses are exploratory and that some associations may not have been detected.

Despite these limitations, our study is important because it provides quantitative data on the relationship between dysphagia and growth and nutritional indicators in pediatric patients with LP, an extremely rare disease. Our findings are hypothesis-generating. Future multicenter prospective studies with prolonged follow-up and multidisciplinary teams should use objective swallowing assessments guided by clinical findings, record dietary intake prospectively, and compare periods with and without active ulcers within the same patients, to clarify the frequency and severity of dysphagia, its relationship with growth, and the underlying mechanisms.

## 5. Conclusions

This cross-sectional study suggests that dysphagia, unlike indicators of oral involvement, may be associated with lower HFA z-scores in pediatric LP. Given the cross-sectional design, small sample, inclusion of siblings (non-independent observations), and clinical definition of dysphagia, the findings should be interpreted cautiously as hypothesis-generating, without causal inference. Swallowing function should be evaluated during follow-up, ideally within a multidisciplinary approach, and larger multicenter prospective studies with standardized objective swallowing assessments are needed.

## Figures and Tables

**Table 1:** Demographic, Anthropometric, and Clinical Characteristics of the Study Population.

Characteristic	*n* (%) or M (SD)
Sex	
Male	20 (66.7)
Age, years	8.44 (4.97)
Parental consanguinity	
Yes	30 (100.0)
Type of consanguinity	
First cousins	28 (93.3)
First cousins once removed	1 (3.3)
Second cousins	1 (3.3)
Affected children in family *	
1	11 (36.7)
2	8 (26.7)
3	4 (13.3)
4	7 (23.3)
Clinical assessment	
HFA z-score	−1.44 (1.07)
WFH/BMI z-score	−0.55 (1.28)
Wasting	2 (6.7)
Stunting	8 (26.7)
Voice change	13 (43.3)
Restricted mouth opening	14 (46.7)
Restricted tongue movement	16 (53.3)
Thick/woody tongue	17 (56.7)
Active oral ulcer	21 (70.0)
Oral ulcer frequency	
Frequent (≤2 weeks)	19 (63.3)
Rare (>2 weeks)	11 (36.7)
Dysphagia	18 (60.0)

Notes: M = mean; SD = standard deviation; HFA = height-for-age; WFH = weight-for-height; BMI = body mass index; *n* = number of patients. Categorical variables are reported as *n* (%); continuous variables are reported as M (SD). * Total number of affected children per family, including non-enrolled siblings aged ≥ 18 years. The 30 patients belonged to 23 families (12 were siblings from five families).

**Table 2:** Laboratory Findings of the Study Population.

Variable	M	SD	Mdn	Range
Glucose, mg/dL	91.41	12.83	87.70	76.00–121.10
Urea, mg/dL	23.28	7.27	23.20	11.70–42.40
Creatinine, mg/dL	0.41	0.15	0.39	0.21–0.86
Albumin, g/L	44.90	2.21	44.85	40.40–50.10
CRP, mg/L	1.90	2.16	0.77	0.18–7.88
ALT, U/L	15.90	8.62	14.65	5.50–46.70
AST, U/L	30.24	9.19	29.05	18.60–58.30
Calcium, mg/dL	9.62	0.37	9.55	9.19–10.75
Magnesium, mg/dL	2.07	0.12	2.06	1.85–2.32
Phosphorus, mg/dL	4.47	0.60	4.56	3.00–5.34
ALP, U/L	195.30	61.96	191.00	42.90–316.00
PTH, pg/mL	33.69	18.78	31.23	17.32–125.20
Vitamin D, ng/mL	30.92	10.83	32.14	4.01–50.35
Hemoglobin, g/dL	11.81	1.74	11.75	8.30–15.70
MCV, fL	73.98	8.35	75.50	56.40–84.40
Iron, µg/dL	48.34	27.34	45.55	12.60–133.20
TIBC, µg/dL	331.91	76.63	325.70	218.50–519.90
Ferritin, ng/mL	34.23	20.97	32.36	7.52–81.43
Vitamin B12, pg/mL	473.78	179.98	414.00	202.60–892.60
Folate, ng/mL	6.94	3.34	6.27	2.94–20.00

Note. M = mean; SD = standard deviation; Mdn = median; ALT = alanine aminotransferase; AST = aspartate aminotransferase; ALP = alkaline phosphatase; CRP = C-reactive protein; TIBC = total iron-binding capacity; PTH = parathyroid hormone; MCV = mean corpuscular volume.

**Table 5:** Spearman’s Rank-Order Correlations Between the Composite Oral Involvement Score and Nutritional, Anthropometric, and Hematological Parameters.

Variable	Spearman’s Rho	*p*
Age, years	0.070	0.712
HFA z-score	−0.143	0.452
WFH/BMI z-score	−0.191	0.312
Albumin	−0.252	0.180
Iron	0.234	0.212
TIBC	−0.351	0.057
Ferritin	0.094	0.623
Vitamin D	−0.111	0.559
Vitamin B12	−0.158	0.405
Folate	0.081	0.672
Hemoglobin	0.003	0.986

Notes: Composite oral involvement score = restricted mouth opening + restricted tongue movement + thick/woody tongue + active oral ulcer (0–4). HFA = height-for-age; WFH = weight-for-height; BMI = body mass index; TIBC = total iron-binding capacity.

## Data Availability

The datasets generated and/or analyzed during the current study are not publicly available but are available from the corresponding author upon reasonable request.

## Abbreviations

The following abbreviations are used in this manuscript:

LP	lipoid proteinosis
ECM1	extracellular matrix protein 1
PAS	periodic acid–Schiff
WHO	World Health Organization
HFA	height-for-age
WFH	weight-for-height
BMI	body mass index
Hb	hemoglobin
MCV	mean corpuscular volume
TIBC	total iron-binding capacity
25(OH)D	25-hydroxyvitamin D
ALP	alkaline phosphatase
PTH	parathyroid hormone
ALT	alanine aminotransferase
AST	aspartate aminotransferase
CRP	C-reactive protein
SD	standard deviation
IQR	interquartile range
FEES	fiberoptic endoscopic evaluation of swallowing
VFSS	videofluoroscopic swallowing study
